# Redo Lung Transplantation After Heart-lung Transplantation

**DOI:** 10.1016/j.atssr.2025.03.008

**Published:** 2025-03-24

**Authors:** Justin C.Y. Chan, Travis C. Geraci, Luis F. Angel, Stephanie H. Chang

**Affiliations:** 1 Department of Cardiothoracic Surgery, NYU Langone Health, New York, New York

## Abstract

We describe the case of a 36-year-old woman who underwent redo lung transplantation AFTER a heart-lung transplant 3.5 years prior. The retransplantation was performed through sequential left posterolateral thoracotomy followed by right anterior thoracotomy, without sternal division and without the use of extracorporeal membrane oxygenation or cardiopulmonary bypass support. The patient was found to have undergone an extensive pericardiectomy at the time of the initial heart-lung transplant. The patient recovered uneventfully and complete healing of the airway anastomosis was demonstrated. This novel technique avoids some potential pitfalls of redo lung transplantation after heart-lung transplant.

In an approximately 30-year period between 1982 and 2013, 3767 heart-lung transplants were reported to the International Society for Heart and Lung Transplantation. In the same period, only 56 (1.5%) lung retransplants after heart-lung transplant occurred.^1^ The most recently available International Society for Heart and Lung Transplantation registry data suggest that approximately 30% of 5-year survivors of heart-lung transplant will develop bronchiolitis obliterans.^2^ Although the use of heart-lung transplantation has significantly diminished in the last decade (due to end-stage pulmonary arterial hypertension primarily being offered double lung transplant^3^), the circumstance of chronic lung allograft dysfunction without corresponding chronic rejection of the cardiac allograft raises the issue of how best to retransplant these patients.

Signed permission from the patient was obtained to share the details of the case. A 36-year-old woman was referred to our hospital with a 2-month history of progressive dyspnea and cough. Three and a half years previously, she underwent heart-lung transplantation for end-stage pulmonary hypertension and secundum atrial septal defect. She was compliant with all medications. Pulmonary function testing showed a severe decline in forced expiratory volume in 1 second (0.6 L) compared with peak post-transplant value (1.79 L). Symptoms did not improve despite antibiotics and steroid pulse. Computed tomography scan was consistent with chronic lung allograft dysfunction, bronchiolitis obliterans syndrome. The function of the transplanted heart was good, with left ventricular ejection fraction 65%, no significant cardiac allograft vasculopathy on left heart catheter, cardiac output 5.7 L/min and pulmonary pressure of 38/15 (mean 24) mm Hg.

The multidisciplinary team discussed her case and she agreed to undergo redo lung transplantation.

Surgery was performed through a combination of left posterolateral thoracotomy and right anterior thoracotomy. This was to optimize exposure of the left hilum due to concern with the cardiac graft overhanging the hilum and adhesions to posterior sternum (Figure 1). A 5 F vascular sheath was placed in the femoral vein and artery to allow for cardiopulmonary bypass/extracorporeal membrane oxygenation cannulation if necessary. Moderate adhesions were encountered and it was noted that a wide pericardiectomy was previously performed during the original transplant surgery (Figure 2). The left lung was implanted, the thoracotomy closed, and patient repositioned supine to implant the right lung through anterior thoracotomy. Care was taken not to devascularize the native bronchial stumps. The implant was performed without cardiopulmonary bypass or extracorporeal membrane oxygenation support. Cold ischemia time was 171 minutes on the left side and 360 min on the right.

**Figure 1:**
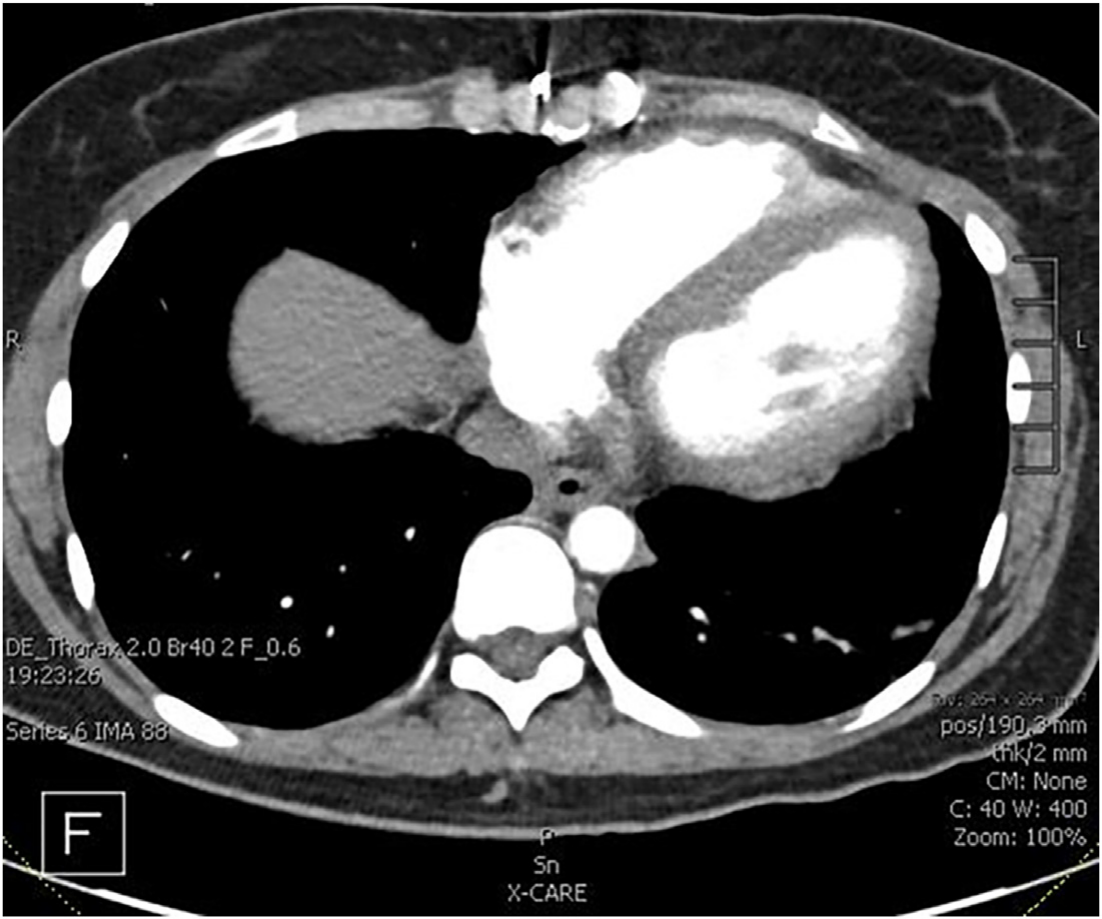
Preoperative computed tomography scan showing broad surface of contact of right and left ventricle to left anterior chest wall.

**Figure 2:**
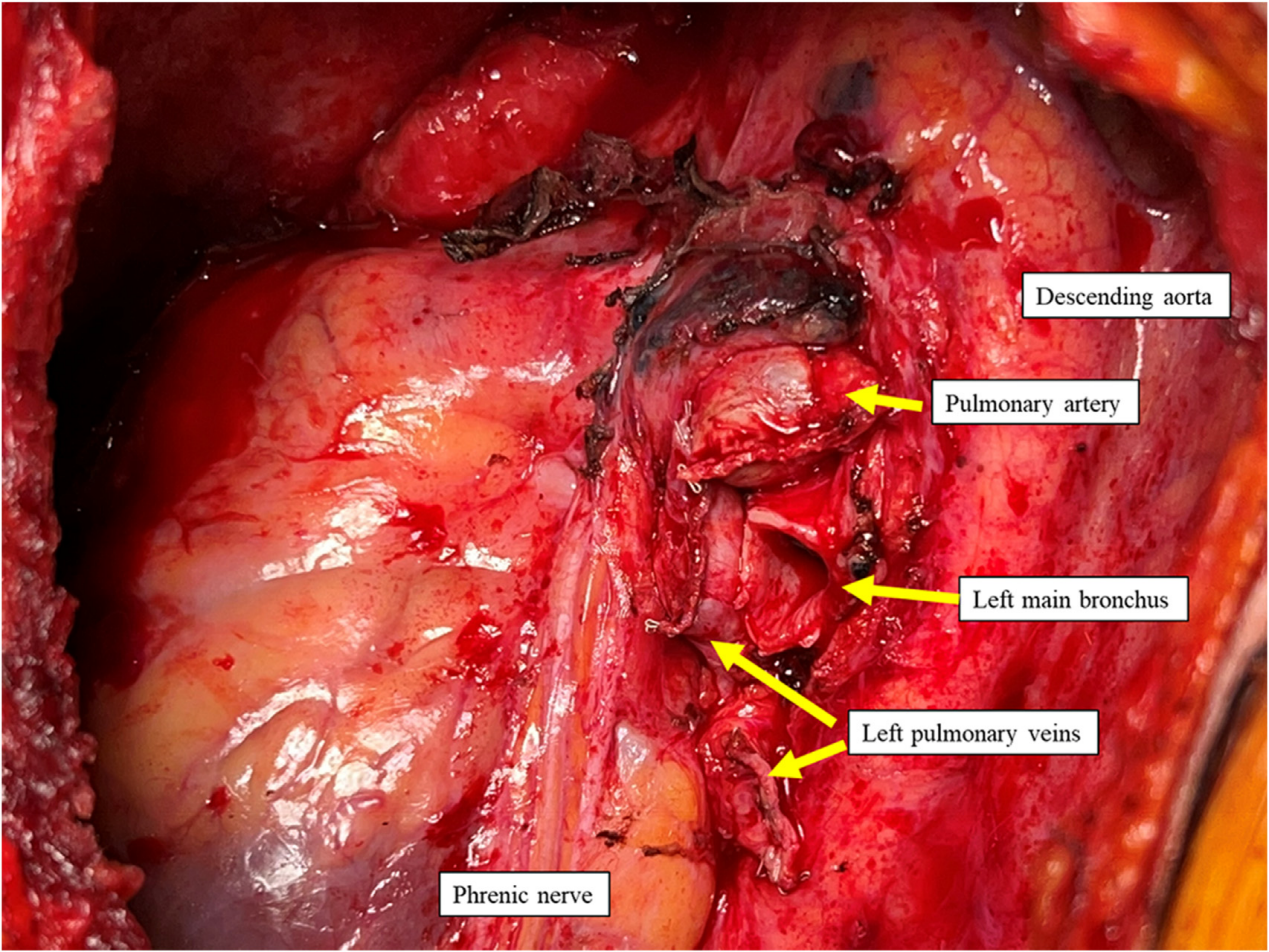
Intraoperative photograph after left pneumonectomy through posterolateral thoracotomy, pericardium widely opened from original transplant.

Postoperative recovery was unremarkable. Bronchoscopy at 6 weeks showed complete healing of bilateral bronchial anastomoses (Figure 3). At most recent follow-up (47 weeks), the patient is doing well with no evidence of rejection and forced expiratory volume in 1 second 1.45 L.

**Figure 3:**
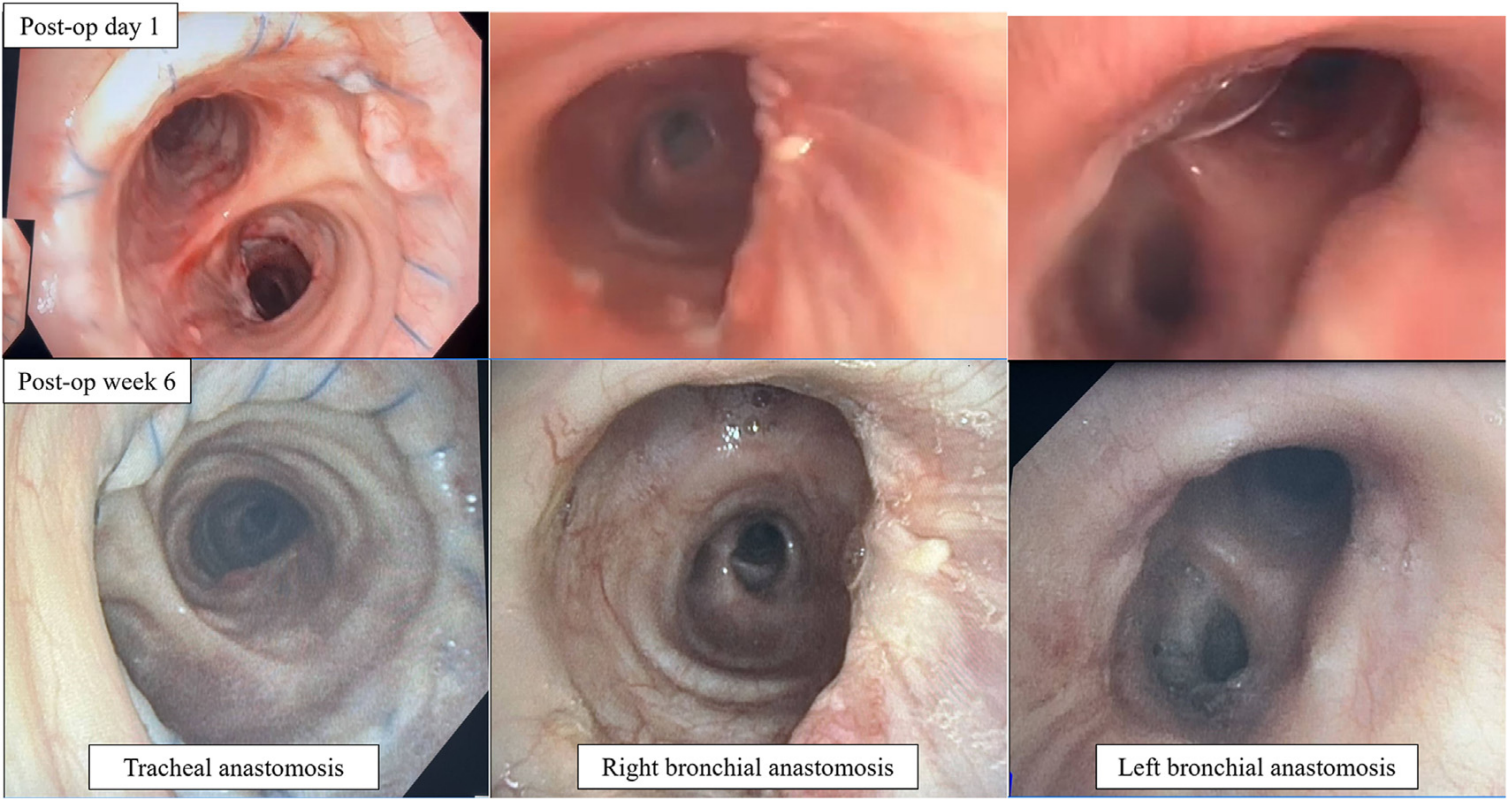
Bronchoscopy immediately postoperatively (post-op) and at 6 weeks showing well-healed donor-to-donor bronchial anastomosis.

## Comment

Redo lung transplant can be a technically challenging procedure. Previous heart-lung transplantation raises unique challenges above that of performing a redo lung transplant procedure alone.

The time between the initial transplant surgery and the reoperation may have implications for the technical difficulty of the surgery (due to adhesions) or patient candidacy. Firstly, early re-transplantation (within 30 days) for primary graft dysfunction has been associated with poor outcomes in both heart^4^ and lung^5^ recipients. The density of adhesions may also be affected by the reoperative interval. However, the technical difficulty of reoperation and adhesions is difficult to accurately predict, even with imaging, and there is some experimental evidence that the potent immunosuppression required for transplantation may reduce the formation of adhesions.^6^

Appropriate patient selection is a crucial factor in determining the success of the procedure. The decision to offer lung retransplant after heart-lung transplant therefore is made carefully on a case-by-case basis, based on the patient’s clinical status and comorbidities, indication for retransplant (chronic lung allograft dysfunction, bronchiolitis obliterans phenotype), cardiac function, expected surgical challenges based on review of imaging and previous operation notes, as well as the experience of the transplant surgeon.

One major surgical concern is the healing of the bronchial anastomosis. Donor-to-donor bronchial anastomosis is feasible and bronchial artery revascularization is not mandatory for healing of the anastomosis^7^; our experience is consistent with this.

The extent of the prior pericardiectomy at the time of initial transplant can complicate the retransplant through adhesions. Early technical reports of heart-lung transplant described wide pericardial excision, leaving the phrenic nerve on a pedicle.^8^ This was the situation in our case, and careful examination of the CT scan, combined with review of the original operation notes or knowledge of local practices, is essential to be able to anticipate the expected anatomy. Identification of the phrenic nerve is crucial to avoid injury and adverse outcomes. In this case, meticulous dissection around the area we expected to find the nerve bilaterally was important. Tracing the complete course of the nerve from the arch of the aorta (or superior vena cava on the right) to the diaphragm allows for proper identification, even with possible abnormal course due to the prior surgery.

Our normal practice is to perform lung transplantation through a bilateral thoracosternotomy (clamshell) incision. To prevent injury to the cardiac allograft, and to minimize the need to use cardiopulmonary bypass or extracorporeal membrane oxygenation, we modified our technique to perform the left lung implant via posterolateral thoracotomy. This did not increase ischemic time notably, and allowed for a sternal-sparing approach to the redo transplantation. This is in contrast to the previous report from Coster and associates,^7^ who utilized a single lumen endotracheal tube, clamshell incision including transverse sternotomy, and cardiopulmonary bypass. We believe the modified approach allows for improved wound healing as well as fewer bleeding complications from cardiopulmonary bypass.

Redo lung transplantation after heart-lung transplantation is both technically feasible and allows for efficient use of donor organs.
